# The impact of macrophage metabolism on idiopathic pulmonary fibrosis development and treatment prospectives

**DOI:** 10.3389/fimmu.2026.1892250

**Published:** 2026-09-16

**Authors:** Guangyan Mu, Shan Chong, Jiulong Li, Hanping Wang, Huan Meng, Qian Xiang, Yimin Cui

**Affiliations:** 1 Department of Pharmacy, Peking University First Hospital, Beijing, China; 2 Institute of Clinical Pharmacology, Peking University First Hospital, Beijing, China; 3 Beijing Key Laboratory of Clinical Pharmacology and Translation of Innovative Drugs, Beijing, China; 4 Department of Pharmacy Administration and Clinical Pharmacy, School of Pharmaceutical Sciences, Peking University, Beijing, China; 5 Chinese Academy of Sciences (CAS) Key Laboratory for Biomedical Effects of Nanomaterials and Nanosafety, Chinese Academy of Sciences (CAS) Center for Excellence in Nanoscience, National Center for Nanoscience and Technology, Beijing, China; 6 Division of Pulmonary and Critical Care Medicine, State Key Laboratory of Complex Severe and Rare Diseases, Peking Union Medical College Hospital, Chinese Academy of Medical Sciences & Peking Union Medical College, Beijing, China; 7 State Key Laboratory of Natural and Biomimetic Drugs, School of Pharmaceutical Sciences, Peking University, Beijing, China

**Keywords:** idiopathic pulmonary fibrosis, immunometabolism, macrophage polarization, metabolic reprogramming, targeted therapy

## Abstract

Idiopathic pulmonary fibrosis (IPF) is a chronic, progressive fibrotic interstitial pneumonia of unknown etiology, in which macrophages serve as central orchestrators of the pathological microenvironment. During IPF progression, the alveolar macrophage pool undergoes a critical compositional shift: tissue-resident alveolar macrophages (TR-AMs) are progressively depleted and replaced by monocyte-derived alveolar macrophages (Mo-AMs) harboring a distinct pro-fibrotic transcriptional and metabolic identity. These populations differ fundamentally in their baseline metabolic programs, and both undergo further reprogramming within the fibrotic niche that sustains myofibroblast activation and extracellular matrix (ECM) deposition. This review systematically examines how metabolic reprogramming across three principal axes (glucose metabolism, lipid metabolism, and amino acid metabolism) drives pro-fibrotic macrophage polarization in IPF. Within each axis, alterations in metabolic flux and intermediate accumulation converge to reinforce the pro-fibrotic phenotype, promote fibroblast activation, and perpetuate ECM deposition. Importantly, these metabolic alterations differ between TR-AM and Mo-AM subpopulations in ways that reflect their distinct ontogenetic origins and predispose Mo-AMs to preferential pro-fibrotic polarization within the fibrotic microenvironment. Building on this mechanistic framework, we systematically categorize pharmacological modulators targeting these metabolic checkpoints and evaluate the integration of advanced drug delivery systems for precision intervention within the alveolar macrophage niche. This review ultimately proposes a therapeutic strategy that co-targets macrophage ontogenetic origin, polarization state, and metabolic reprogramming as an interconnected axis, offering new perspectives for re-educating macrophages to attenuate fibrotic progression in IPF.

## 1 Introduction

Interstitial lung disease (ILD) is a series of heterogeneous diffuse lung diseases. Idiopathic pulmonary fibrosis (IPF), the most common ILD, is a chronic, progressive, fibrotic interstitial pneumonia of poor prognosis and unknown etiology (1). The typical histological features of IPF contain subpleural fibrosis and microscopic honeycombing, less-fibrotic central lung tissue, and fibroblast foci (2). Long-term inflammatory responses are important risk factors for fibrotic events that alter cell function and stimulate fibroblasts to transform into myofibroblasts (3). In addition to a history of pulmonary diseases like tuberculosis, pneumonia or chronic obstructive pulmonary disease, older age and oxidant stress are prominent risk factors for IPF (4, 5). Multiple pathways related to metabolism in common biochemical processes of aging have been identified in IPF, including loss in the homeostasis of proteostasis, dysregulated nutrient sensing, mitochondrial dysfunction, etc (6, 7).

Mitochondrial dysfunction and metabolic reprogramming are found in lung epithelial cells, fibroblasts and macrophages during IPF, which promote low cellular resilience and increase susceptibility to activation of profibrotic responses and lead to fibrotic development (8). Although current antifibrotic therapies, such as pirfenidone and nintedanib, have demonstrated efficacy in decelerating the annual decline of lung function, they do not halt progression in the majority of patients (9). This therapeutic plateau suggests that predominantly targeting fibroblast activation overlooks the fundamental drivers of the persistent fibrotic niche. Emerging evidence indicates that the intractable nature of IPF is driven by a profound metabolic rewiring of the innate immune landscape, particularly macrophages, which sustains a pro-fibrotic microenvironment through the loss of immune homeostasis. While the recent clinical success of the PDE4B inhibitor nerandomilast highlights the potential of modulating intracellular signaling for immunomodulation, the precise metabolic checkpoints that govern macrophage plasticity remain poorly understood (10).

Macrophages induce inflammation and play different phagocytic and immune roles in the development of IPF according to their subtypes, however, the specific mechanisms remain unclear (11). The therapeutic strategies that focus on regulation of macrophage-related chemokines, cytokines and the networks may be a new approach of pharmacotherapy for IPF (12). Macrophages have unique metabolic characteristics related to their functional state, which is known as metabolic reprogramming, including glycolysis, tricarboxylic acid (TCA) cycle, mitochondrial oxidative phosphorylation (OXPHOS), fatty acid oxidation (FAO), arginine metabolism, glutamine metabolism, etc (13). There are metabolic differences between macrophage populations activated by different sources or stimulation, and they may play different immune roles (14–16). In this review, we first discuss macrophage inflammation in the context of IPF and the different intracellular metabolic pathways that regulate macrophage polarization and function during this process. At the same time, we reviewed the effects and mechanisms of macrophage-related metabolic reprogramming targets and pathways on IPF. With a view to providing references for mechanism research and new drug development.

## 2 Inflammation and macrophages dysregulation in idiopathic pulmonary fibrosis

Fibrosis is the ultimate common endpoint of multiple chronic inflammatory diseases. Chronic inflammation is a major feature of IPF, driven by innate and adaptive immune responses. Crosstalk of fibroblasts and immune cells, such as macrophages and neutrophils, is a key step in promoting the wound healing response, e.g. producing and secreting growth factors and cytokines to induce fibrosis (17, 18). Macrophages are the first line of defense against pathogens and foreign bodies in the lungs, and play an important role in maintaining tissue homeostasis. Macrophages are the major regulators of lung tissue repair, which aggravate the inflammatory response after lung injury and then transit to a healing phenotype to drive the development of pathological fibrosis when the disorder persists (19).

The main types of macrophages in the lung are alveolar macrophages (AMs) and interstitial macrophages (IMs). AMs reside in the alveolar lumen of the lungs and may originate from fetal macrophages during embryonic hematopoiesis, which are responsible for pathogen clearance and the release of inflammatory mediators (20). Circulating monocytes serve as a fresh source of monocyte-derived alveolar macrophages (Mo-AMs) to enhance the AMs pool during IPF (21). IPF patients have more AMs and increased TGF-β (Transforming Growth Factor-β) secretion compared with healthy individuals, which has been recognized as one possible pathogenic factor of lung fibrosis (22). It was found that the expression of fibrotic genes such as *IL1RN* (encoding IL-1RA) and *CHI3L1* (encoding chitinase 3-like protein 1) in AMs (predominantly Mo-AMs) with IPF fibrosis were higher than those in healthy lungs (23). Besides, Westphalen et al. (24) found specific non-sessile tissue-resident alveolar macrophages (TR-AMs) that transmit immunosuppressive signals directly to neighboring epithelial cells to protect the lungs from tissue-damaging inflammation. IMs exist in lung parenchyma and is involved in the maintenance of immune homeostasis of respiratory system (25). The abilities to phagocyte granules or produce reactive oxygen species (ROS) and chemotactic complement were lower in IMs than that to AMs. But IMs express more MHC-II (encoding HLA-DR) to fulfill their antigen-presenting roles (26). Limited information is available on IMs’ participation in IPF.

Activated macrophages show metabolic changes in mechanisms regulating immune effects. It is generally believed that the role of macrophages in IPF is manifested in the pro-inflammatory and anti-fibrotic properties of classically activated M1 macrophages, as well as the anti-inflammatory, pro-fibrotic and tissue regeneration properties of alternatively activated M2 macrophages. After activation by lipopolysaccharide (LPS) and IFN-γ, the original M0 or polarized M2 macrophages differentiated into M1 macrophages (27). Under the stimulation of inducible nitric oxide synthase (iNOS), M1 macrophages produce reactive nitric oxide (NO) and a variety of pro-inflammatory cytokines and chemokines, such as IL-1β, IL-12, IL-23, CCL2, CXCL10 and TNF-α. In particular, factors of CXCL10 and matrix metalloproteinase (MMPs) can promote matrix degradation and inhibit fibrosis (28).

Generally, M2 macrophages are the most representative phenotype in the progression of IPF. It will lead to fibroblasts activation and extracellular matrix (ECM) deposition, meanwhile to secret cytokines and chemokines including IL-4, IL-13 and CCL18, involving in triggering pulmonary fibrosis (29–31). Furthermore, transcription factors and other intracellular proteins such as tuberous sclerosis complex 1 (TSC1) (32), stress-responsive activating transcription factor 7 (ATF 7) (33), STIP1 homology and U-Box containing protein 1 (STUB1) (34), ten eleven translocation (Tet) methylcytosine dioxygenase (Tet2) (35), interferon regulatory factor 4 (IRF4) (36), peroxisome proliferator-activated receptor gamma (PPARγ) (37) and Krueppel-like factor 4 (KLF-4) 4 (38) are also involved in the polarization of M2 macrophages. M2 phenotype is further divided into four subtypes according to different stimulating factors: M2a, M2b, M2c and M2d (39). The M2a refers to trauma repair macrophages that are produced in response to IL-4, IL-10, IL-13, and TGF-β (40). M2a macrophages secrete chemokines CCL17, CCL18 and CCL24, which are involved in tissue repair (41, 42). M2b are known as regulatory macrophages, which are induced by LPS and IL-1R ligand, and increase the expression of IL-1, IL-10 and TGF-β (43). The inability of M2c cells to polarize towards M1 is known as the acquired deactivation of macrophages. M2c cells are induced by IL-10, TGF-β and glucocorticoids, secrete high levels of IL-10 and TGF-β, which are involved in regulating immunosuppressive response and tissue remodeling (44). M2d cells are monocyte-derived macrophages known as tumor-associated macrophages (TAMs), which are mainly induced by autocrine tumor-related factors. M2d phenotype highly express cytokines and chemokines, including IL-6, IL-10, TGF-β, CXCL 10, CXCL 16 and CCL 5, and are involved in promoting angiogenesis, matrix remodeling and adaptive immunosuppression processes (45, 46). A recent study has also identified a unique pro-fibrotic M2-like profile in macrophages from a fibrotic mouse model that is distinct from well-defined M1 or any M2 subtype (47).

While the above M1/M2 frame provides a useful classification of macrophages functionality, especially in terms of cytokine profiles and effector mechanisms, it only represents one dimension of macrophage heterogeneity in IPF. The ontogenetic origin of macrophages is an equally critical dimension which governs the intrinsic metabolic programming in macrophages and determines their polarization propensity within the fibrotic microenvironment (as summarized in **Figure 1**).

**Figure 1:**
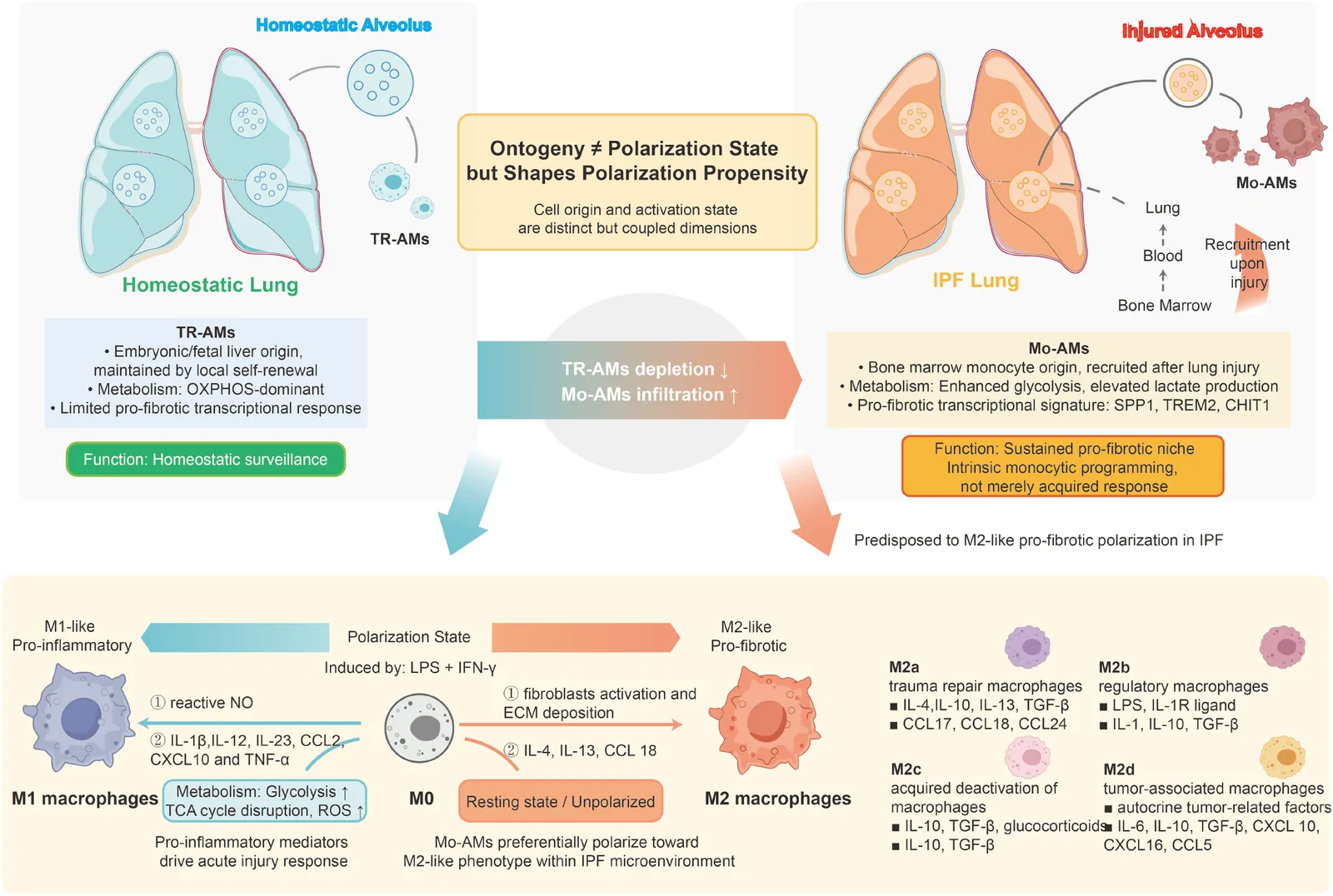
Two-dimensional framework of macrophage heterogeneity in idiopathic pulmonary fibrosis (IPF). Macrophage identity and function in IPF are governed by two distinct but coupled dimensions: ontogenetic origin and polarization state. Under homeostatic conditions, the AMs pool is dominated by TR-AMs, which originate from embryonic precursors and are maintained by local self-renewal. TR-AMs rely predominantly on OXPHOS and exhibit a limited pro-fibrotic transcriptional response. During persistent lung injury in IPF, TR-AMs undergo progressive depletion and are replaced by Mo-AMs recruited from circulating monocytes. Mo-AMs harbor a distinct intrinsic metabolic and transcriptional program characterized by enhanced glycolytic flux, elevated lactate production, and a pro-fibrotic gene signature including*SPP1*, *TREM2*, and*CHIT1*. Macrophage polarization spans a functional continuum from the classically activated M1-like pro-inflammatory phenotype, that is driven by LPS and IFN-γ and characterized by secretion of IL-1β, IL-12, IL-23, TNF-α, CCL2, and CXCL10, through the resting M0 state, to the alternatively activated M2-like pro-fibrotic phenotype, which promotes fibroblast activation and extracellular matrix (ECM) deposition via IL-4, IL-13, TGF-β, and CCL18. M2 macrophages are further classified into four functional subtypes: M2a (trauma repair), M2b (regulatory), M2c (acquired deactivation), and M2d (tumor-associated). Critically, Mo-AMs harbor an intrinsic pro-fibrotic metabolic program that predisposes them to preferential M2-like polarization within the IPF microenvironment, such that the progressive replacement of TR-AMs by Mo-AMs represents a dual shift in both population composition and functional programming that collectively sustains the pro-fibrotic niche. This two-dimensional framework highlights that therapeutic strategies targeting polarization state alone may be insufficient without simultaneously addressing the upstream ontogenetic determinants of macrophage behavior.

Under homeostatic conditions, the AMs pool is dominated by tissue-resident AMs (TR-AMs), which originate from embryonic precursors (yolk sac and fetal liver) and are maintained through local self-renewal (20). TR-AMs are metabolically adapted for sustained surveillance and homeostatic functions, primarily utilizing OXPHOS and exhibiting a limited pro-fibrotic transcriptional response even under mild inflammatory stimuli. In contrast, during persistent lung injury such as that occurring in IPF, TR-AMs undergo progressive depletion and are replaced by Mo-AMs recruited from circulating monocytes (21, 48). Single-cell RNA sequencing data have revealed that Mo-AMs in IPF lungs harbor a distinct transcriptional and metabolic identity, which is characterized by enhanced glycolytic flux, elevated lactate production (49), and a pro-fibrotic gene signature that includes SPP1, TREM2, and CHIT1. It is not merely an acquired response to local stimuli but reflects their intrinsic monocytic programming (23, 50, 51).

Importantly, the two dimensions of polarization state and cell origin are distinct but coupled. Mo-AMs harbor an intrinsic pro-fibrotic metabolic program that predisposes them to preferential M2-like polarization within the IPF microenvironment. The progressive replacement of TR-AMs by Mo-AMs therefore represents a dual shift in both population composition and functional programming that collectively sustains the pro-fibrotic niche. This distinction has direct implications for therapeutic targeting, as interventions aimed at polarization state alone may fail to address the upstream ontogenetic drivers of the persistent fibrotic program (48).

## 3 Alterations on macrophages metabolism during idiopathic pulmonary fibrosis

Macrophages depend on metabolic pathways to provide optimal energy reserves for survival and function like most immune cells. Macrophages activation and cytokine production are closely related to energy production. Metabolic adaptation is critical for altering the macrophage response in a specific inflammatory environment, affecting the polarization state of macrophages. Changes in the polarization state and function of macrophages are usually accompanied by metabolic reprogramming, and the accumulation of metabolites in turn regulates the process of their polarization. The role of metabolic reprogramming in tumorigenesis and development has been well-established; however, more and more metabolic events related to macrophage polarization in IPF have been reported, which has become a new perspective of research attention (52).

Beyond their roles in bioenergetic support, metabolic intermediates in macrophages have emerged as potent signaling ligands that govern transcriptional and epigenetic landscapes during the progression of IPF. This paradigm shift, often termed ‘metabolic signaling,’ suggests that metabolites no longer serve merely as fuel; instead, they function as rheostats that sense microenvironmental changes and directly instruct macrophage functional identity through post-translational modifications and stabilization of key transcription factors (53).

### 3.1 Changes in glucose metabolism

Glycolytic transition appears to be a key metabolic switch in macrophages under fibrotic conditions. The metabolic characteristics of M1 macrophages are greatly different from M2 macrophages. The glycolysis reaction promotes the macrophages polarize toward M1 subtype, which was manifested by the breakdown of TCA cycle and the damage of mitochondrial OXPHOS, following with the increasing production of ROS. At this point, TCA cycle is disrupted at both citrate and succinate, leading to the accumulation of certain endogenous metabolites with immunoregulation effects, such as succinate and itaconate, that in turn play a pro-inflammatory role (54) (**Figure 2**).

**Figure 2:**
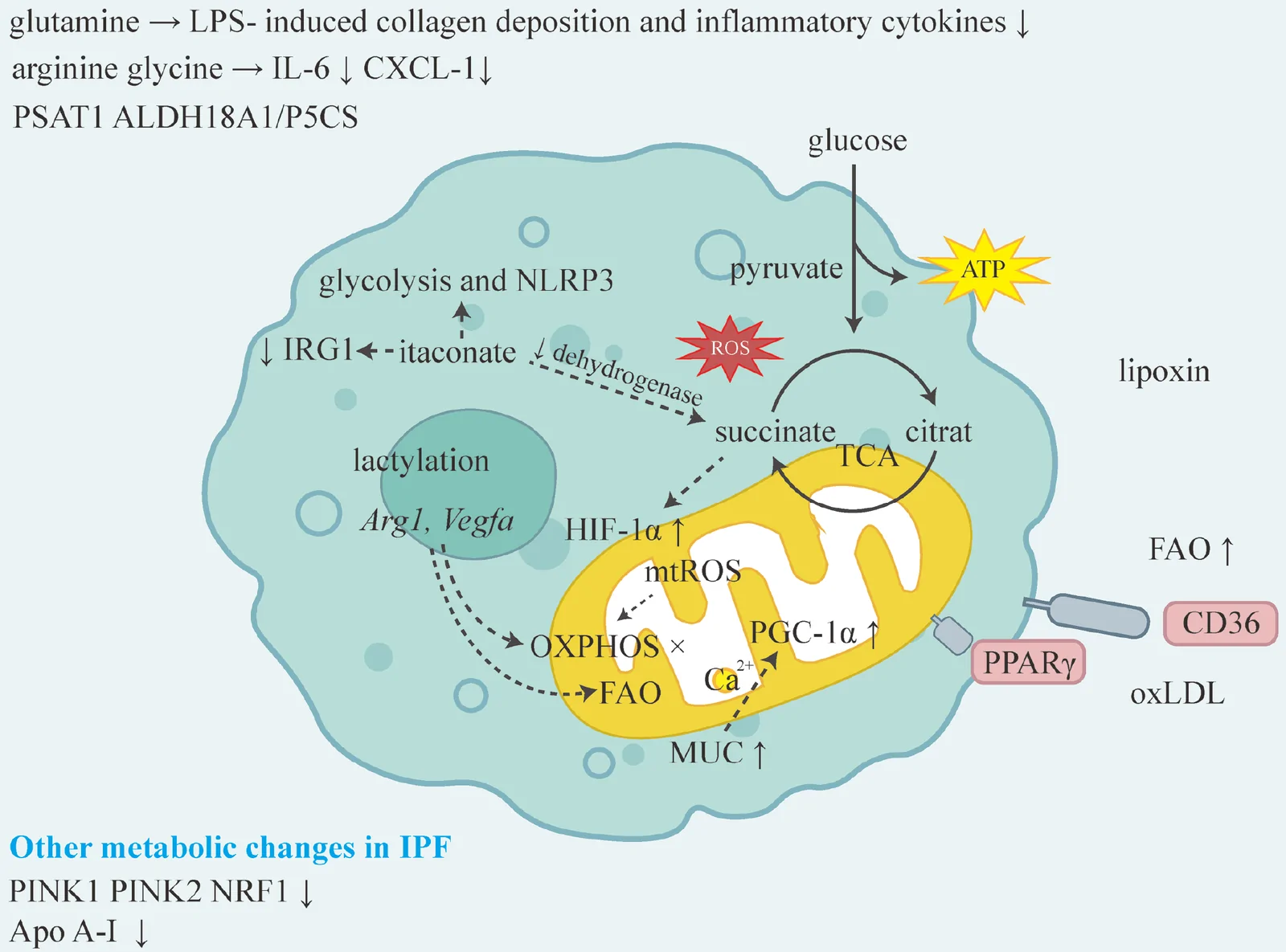
Key glucose and lipid metabolisms in macrophages during IPF.

Succinate dehydrogenase acts on succinate to produce fumarate in the TCA. Succinate in macrophage cytoplasm acts as a signaling molecule that increases the expression of hypoxia-inducible factor 1α (HIF-1α) and leads to increased inflammation through IL-1β induction (55). It was proved that the regulation of macrophage polarization by glucose metabolism reprogramming intermediates is of great significance for improving pulmonary vascular remodeling and acute lung injury (56, 57). Whether a comparable pattern of succinate accumulation occurs in alveolar macrophages within the fibrotic lung microenvironment remains to be directly verified. The downstream signaling consequences of succinate accumulation are addressed in Section 4.1.

Itaconate, also known as immune response gene 1 (IRG1), is highly expressed in mammalian macrophages during inflammation (58). As a key counter-regulatory metabolite derived from cis-aconitate, itaconate accumulates in response to TCA disruption and exerts broad inhibitory effects on pro-inflammatory metabolic flux, including SDH inhibition and suppression of aerobic glycolysis (59). The immunomodulatory function of itaconate exhibits cell-type- and tissue-microenvironment-specificity. In TR-AMs, endogenous itaconate promotes pro-inflammatory responses, rather than the previously established anti-inflammatory function, by inhibiting succinate dehydrogenase (SDH) and enhancing mitochondrial electron transport chain complex I (ETC-CI) activity, thereby robustly facilitating NOD-Like Receptor Thermal Protein Domain Associated Protein 3 (NLRP3) inflammasome activation and the release of pro-inflammatory cytokines, including IL-1β, IL-6, and IL-12. *In vivo* experiments demonstrate that itaconate exacerbates LPS-induced acute lung injury (ALI), whereas IRG1 knockout improves survival rate. In contrast, its derivatives—DMIA and 4-OI—exert anti-inflammatory effects in AMs (60). In chronic fibrotic conditions such as IPF, itaconate instead functions as an endogenous antifibrotic mediator to restrain maladaptive fibrotic remodeling rather than to resolve acute immune activation. IRG1 expression was observed reduced in isolated AMs of IPF patients, which is further proved by the aggravated pulmonary fibrosis in a IRG1 KO bleomycin mice model (61). Recent studies have highlighted itaconate as a potential intervention target for pulmonary diseases such as asthma, IPF and respiratory infections (62). Given that the immunomodulatory effect based on itaconic acid is tissue type and microenvironment-specific, it is essential to conduct a comprehensive assessment of its function in resident macrophages in different tissues before its clinical application.

M2 macrophages mainly rely on OXPHOS reaction for energy supply. M2 macrophages can promote the expression of components in the electron transport chain and drive pyruvate into TCA, prompting the TCA cycle combines with OXPHOS to produce adenosine triphosphate (ATP) (13). Certain chronic diseases involving IL-4 signaling, such as parasitic infections, allergies, asthma, etc., may be related to the persistent maintenance of OXPHOS status by M2 macrophages, meanwhile chronic inflammatory stimulation may be related to the inhibition of aerobic glycolysis of macrophages as well (63). It is noteworthy that Xie et al. (47) has found that unlike typical IL-4-induced macrophages, fibrosis-like M2 cells in fibrotic AMs were not depend on fatty acid oxidation and synthesis(FAO and FAS)or lipolysis, but instead on glycolysis. They observed augmented glycolysis and enhanced glucose consumption in AMs (predominantly Mo-AMs) from mouse lungs treated with bleomycin.

Pathological lactate accumulation in the fibrotic microenvironment has been identified as a key driver of M2 macrophage polarization and fibroblast activation (64). In the fibrotic lung microenvironment, activated myofibroblasts undergo a metabolic shift toward enhanced glycolysis, resulting in substantial lactate secretion into the extracellular space. This pathological lactate accumulation directly promotes fibroblast proliferation, differentiation, and collagen synthesis, thereby aggravating fibrosis remodeling (65, 66). Beyond its extracellular effects, lactate functions as a core paracrine signaling molecule rather than passive metabolic waste: it is taken up by neighboring AMs and drives their polarization toward a profibrotic phenotype (67). This process is mechanistically linked to lactylation, a recently discovered posttranslational modification. Under normal physiological conditions, 724 lysine lactylation sites in 451 proteins have been identified in human lungs (68). In macrophages, histone lactylation (Kla) serves as a unique epigenetic switch during the late stage of M1 polarization, inducing the expression of homeostatic genes with M2-like repair functions and thereby facilitating the transition from acute inflammation to stable tissue repair (69). In the pathological context of IPF, however, this lactylation machinery is co-opted by the fibrotic microenvironment. Collectively, these findings establish lactate metabolism as a central and therapeutically targetable node in IPF progression, coordinating both structural remodeling and immune reprogramming within the fibrotic niche.

Mitochondrial dysfunction and oxidative stress are significant features of prefibrotic reactions and are closely related to metabolic reprogramming. Gu et al. (70) found a prominent reduction of mitochondrial reactive oxygen species (mtROS) and FAO in macrophages in mice harboring a conditional expression of dominant-negative MCU, showing a protective effect of IPF. They also revealed that IPF lung macrophages have increased mitochondrial calcium uniporter (MCU) expression and mitochondrial calcium. All these suggested the contribution of macrophage MCU-mediated metabolic reprogramming in fibrotic repair after lung injury. Moreover, Tsitoura et al. (71) reported that marked morphological defects and impaired transcription are found in AMs from IPF patients, manifested as significantly increased mtROS, which might be associated with age, decreased pulmonary function and also reduced OXPHOS related gene expression.

The important point is that several mechanistic conclusions regarding the reprogramming of macrophage glycolysis in this section are largely derived from studies conducted in non-lung contexts. Currently, there is still very limited direct evidence of the glycolytic conversion in human alveolar macrophages in the lungs of IPF. Therefore, the applicability of these mechanisms in the biology of lung macrophages should be treated with caution, and direct verification through IPF-specific experimental models is required, such as patient-derived alveolar macrophages and humanized lung organoid systems.

### 3.2 Changes in lipid metabolism

Fatty acids (FAs) are transported to mitochondria by carnitine and produce ATP through β-oxidation, which has a significant impact on the metabolic reprogramming of macrophages. In alveolar macrophages, this transport is facilitated by carnitine palmitoyl transferase 1 (CPT1), which conjugates fatty acyl-CoA to carnitine for translocation across the inner mitochondrial membrane, where carnitine palmitoyl transferase 2 (CPT2) regenerates fatty acyl-CoA for subsequent β-oxidation, yielding NADH, FADH2, and acetyl-CoA that feed the TCA cycle and electron transport chain (72, 73). This oxidative capacity becomes a central axis around which macrophage fate and fibrotic progression are organized in the context of IPF.

Increased FAO levels were found in lung macrophages during IPF lungs. In bleomycin-induced pulmonary fibrosis, AMs show increased FAO that is dependent on the mitochondrial calcium uniporter and PPARγ coactivator 1-alpha (PGC-1α), indicating that mitochondrial calcium handling and transcriptional co-activation converge to amplify FAO in the pro-fibrotic state (70). FAs provide energy to support macrophage polarization and FAO produces large amounts of ATP, which is thought to promote M2 macrophages polarization. Importantly, sustained FAO is associated with prolonged macrophage survival, as reliance on this pathway rather than glycolysis extends cellular lifespan and may thereby maintain a pro-fibrotic macrophage population within the fibrotic niche (72, 74, 75). This metabolic bias toward FAO over glycolysis has been proposed to impair homeostatic macrophage turnover, potentially contributing to the persistence of pro-fibrotic signaling and collagen deposition rather than injury resolution (75). Altered fatty acid composition disrupts the homeostasis of type II alveolar epithelial cells, which may participate in pathogenesis of pulmonary fibrosis by promoting the activation of M2 macrophages (70). Macrophages can also enhance fibrotic activity by altering FA metabolism of fibroblasts in IPF, suggesting that lipid metabolic crosstalk between macrophages and stromal cells represents an additional pro-fibrotic axis (76).

The heightened energy demand in pro-fibrotic macrophages is accompanied by dysregulated lipid uptake, which drives pathological lipid accumulation. High-fat diet rich in palmitic acid has been shown to induce fibrosis by increasing endoplasmic reticulum stress and cell death in lung epithelial cells (77). A similar process (palmitic acid promotes endoplasmic reticulum stress) also appears to occur in macrophage (78). CD36 is a widely expressed transmembrane fatty acid receptor. After entering the cell FAs pool, CD36 can be esterified with glycerol or sterol skeleton, and stored in the form of triglycerides in lipid droplets, or through FAO to produce energy. Of note, CD36 expression is not subject to negative feedback regulation by intracellular cholesterol accumulation, in contrast to the classical low-density lipoprotein receptor (LDLR) pathway; furthermore, oxLDL-driven activation of PPARγ further upregulates CD36 expression through a positive feedback loop, collectively rendering this receptor a significant contributor to uncontrolled lipid accumulation in macrophages (79–81). CD36 deficiency can reduce levels of Th2 cytokines that promote fibrosis, including IL-9, IL-4 and IL-13, and inhibit pulmonary fibrosis (82).

A more direct link between lipid accumulation and pulmonary fibrosis is provided by evidence from the fibrotic lung microenvironment itself. Bleomycin-induced damage to alveolar type II (AT2) cells causes release of stored lipids and cholesterol into the alveolar space, leading to lipid accumulation and a pro-fibrotic response in alveolar macrophages (83). Pulmonary foam cells increased significantly in silicosis patients, while the levels of intracellular oxidized low-density lipoprotein (oxLDL) and lipids clearance receptors like CD36 and PPARγ increased as well (84). *In vitro* studies confirm that oxLDL uptake promoted the transformation of silica dust-driven rat lung macrophages into foam, and TGF-β production was significantly inhibited after antagonizing CD36 or PPARγ. An analogous mechanism has been described in liver fibrosis, where Kupffer cells absorb modified cholesterol-rich lipoproteins through scavenger receptors (SRs), leading to cholesterol accumulation and cell activation; inactivation of the SR pathway reduced macrophage and neutrophil infiltration, providing a mechanistic parallel that may inform our understanding of macrophage lipid loading in the lung (85). Lipid-laden macrophages in the fibrotic niche are also sustained by TREM2 signaling, which recognizes sphingolipid ligands and promotes survival of this foam-cell population in both murine and human fibrotic lung, and blockade of TREM2 or its sphingolipid ligands has been proposed as a strategy to attenuate macrophage-driven fibrosis (86–88). Furthermore, ceramide and sphingosine, products of sphingolipid catabolism, have been shown to inhibit the efferocytic activity of alveolar macrophages, a function critical for the resolution of injury (89).

When lipid uptake exceeds the capacity for efflux, cholesterol accumulates and foam cell formation ensues. Cholesterol efflux therefore represents a critical counter-regulatory mechanism. Macrophages have been shown to regulate FAs synthesis under the control of liver X receptors (LXRs) α and β, which autonomously regulate its function and the function of surrounding parenchymal cells in an autocrine/paracrine manner.

Apolipoprotein A-I (ApoA1) mediates the outward transport of cholesterol from cells. Cholesterol will accumulate in alveolar macrophages after ApoA1 reduction, resulting in an increase in the number of foam cells (90). Further studies in mice showed that intranasal administration of ApoA1 decreased bleomycin-induced increases in number of inflammatory cells and collagen deposition in a dose-dependent manner, so as to slow lung injury and fibrosis development (91). Together, these findings suggest that impaired reverse cholesterol transport, driven by reduced ApoA1 and dysregulated LXR signaling, creates a permissive environment for foam cell accumulation and sustained pro-fibrotic macrophage activation.

Beyond structural lipids, alveolar macrophages generate a spectrum of bioactive lipid mediators that actively shape the inflammatory and fibrotic milieu. Lipoxins (LX) are important endogenous lipids inflammatory matrix that regulate different types of macrophages in pulmonary fibrosis. More broadly, alveolar macrophages metabolize phospholipids derived from lung surfactant into eicosanoids via the lipoxygenase and cyclooxygenase pathways; alveolar macrophages highly express 5-lipoxygenase, which synthesizes leukotrienes including leukotriene B4 (LTB4), a potent chemoattractant for neutrophil recruitment, and cysteinyl leukotrienes (LTC4, LTD4, and LTE4), which contribute to bronchoconstriction (92). Prostaglandin E2 (PGE2), another arachidonic acid-derived eicosanoid produced by alveolar macrophages, is linked to efferocytosis and promotes tissue repair; reduced PGE2 production by alveolar macrophages has been associated with inflammatory lung diseases (92–94). In contrast to these pro-inflammatory mediators, lipoxins serve as endogenous resolution signals. Aspirin-triggered LX synthetic analogues lipoxin A4 (LXA4) have been reported to have a therapeutic power to reduce cytokine production and restore M2 macrophage populations in bleomycin-induced pulmonary fibrosis (95, 96). Besides, LXA4-FPR2 signaling can regulate radiation-induced pulmonary fibrosis by crosstalk with TGF-β/Smad pathways (97). These findings highlight the dual role of macrophage-derived lipid mediators: while leukotrienes amplify early inflammatory recruitment, lipoxins and PGE2 promote resolution and repair, and their imbalance in IPF may tip the scale toward persistent fibrotic activation.

However, it must be acknowledged that most of the evidence related to lipid metabolism comes from *in vitro* experiments using BMDMs or peritoneal macrophages, supplemented by support from macrophages in atherosclerotic plaques or liver fibrosis. Alveolar macrophages occupy a unique tissue-resident site, with a microenvironment characterized by abundant surfactants, low glucose, and hypoxia, which is fundamentally different from the environments of peritoneal or liver macrophages. In the steady-state lung, alveolar macrophages rely predominantly on OXPHOS and FAO rather than glycolysis, a metabolic profile that is dependent on PPARγ and that is disrupted upon removal of these cells from their native niche (98–101). Therefore, in the context of IPF, the quantitative contribution and directional effects of these mitochondrial metabolic pathways may vary. Future studies should employ single-cell metabolomics to analyze patient-derived alveolar macrophages and combine spatial transcriptomics of fibrotic lung tissue to directly verify the validity of these inferred mechanisms in the pulmonary environment.

### 3.3 Changes in amino acid metabolism

Arginine and glutamine are the two main amino acid substrates for amino acid metabolism in IPF-related macrophages. Their breakdown products jointly enter the TCA and jointly determine the pro-fibrotic or pro-inflammatory effects of macrophages.

Arginine metabolism through the arginase-1(ARG1) pathway has been implicated in pro-fibrotic macrophage function. ARG1 converts arginine to ornithine, which is subsequently metabolized to proline, providing substrate for collagen synthesis in fibroblasts and thereby directly contributing to ECM deposition. This mechanism has been directly demonstrated in models of pulmonary fibrosis: ARG1-expressing macrophages drive pro-fibrotic metabolic reprogramming of fibroblasts through the ornithine–proline axis (102). The expression of ARG1 in lung macrophages is induced by the type 2 cytokines IL-4 and IL-13, a cytokine milieu that is also present in fibrotic lung tissue, further supporting the relevance of this pathway to fibrotic pathogenesis (103).

Ornithine generated by ARG1 does not feed exclusively into collagen synthesis, but also serves as the entry point for the polyamine biosynthesis branch. The polyamine pathway represents an alternative branch of ornithine metabolism, through which ornithine is converted to spermidine and spermine. Notably, spermidine has been shown to attenuate bleomycin-induced pulmonary fibrosis in mice through mechanisms involving the induction of autophagy and inhibition of endoplasmic reticulum stress-induced cell death (104), suggesting that the arginine–polyamine axis exerts bidirectional regulatory effects on fibrotic progression. The flux directed toward proline supports collagen deposition, whereas flux toward polyamines may engage protective autophagy. Whether this branch-point distribution is dynamically regulated in IPF macrophages remains to be established.

A distinct and functionally opposing branch of arginine metabolism involves iNOS-mediated generation of nitric oxide (NO). IFNγ derived from T lymphocytes and natural killer cells stimulates iNOS expression in macrophages, and the resulting NO production serves both anti-bacterial and pro-inflammatory functions (72). Increased iNOS expression and NO production by alveolar macrophages have been documented in COPD and pulmonary fibrosis (105). Because ARG1 and iNOS compete for the same arginine substrate, the relative dominance of each enzyme determines the directional partitioning of arginine flux. In the pro-fibrotic macrophage, ARG1 predominance diverts arginine toward the ornithine–proline axis, while suppressing the iNOS–NO arm that would otherwise support inflammatory resolution. Although direct quantification in IPF macrophages is lacking, the ARG1-iNOS axis provides a mechanistic framework for understanding arginine flux directionality.

Alongside arginine, glutamine represents the second major amino acid substrate whose metabolism is dysregulated in the IPF macrophage niche. Collagen synthesis by lung fibroblasts requires glutamine metabolism, glutamate-consuming enzymes phosphoserine aminotransferase 1 (PSAT1) and aldehyde dehydrogenase 18A1 (ALDH18A1)/Δ1-pyrroline-5-carboxylate synthetase (P5CS) are required for the production of collagen by lung fibroblasts. New glycine production requires PSAT1, while new proline production requires ALDH18A1/P5CS (106). This finding suggests glutamine as a shared metabolic resource between macrophages and fibroblasts within the fibrotic niche, raising the question of whether macrophage glutamine utilization and fibroblast glutamine utilization are in competition or coordination.

Beyond its role in supporting fibroblast collagen synthesis, glutamine also directly regulates macrophage polarization state (107). Glutamine synthetase (GS) activity is the intermediary of M2-like macrophages in promoting angiogenesis, immunosuppression and metastasis. GS inhibitors can skew M2-polarized macrophages toward M1-like phenotypes, characterized by decreased intracellular glutamine, together with increased succinate and glycolysis (108). This pharmacological evidence positions GS as a potential therapeutic node that GS inhibition can redirect macrophage polarization away from the pro-fibrotic M2 state by modulating intracellular glutamine availability. The possibility of targeting GS in the treatment of tumor metastasis has been mentioned, which also provides a reference for IPF.

A third dimension of glutamine’s regulatory role in macrophages operates through epigenetic reprogramming via the α-KG axis. Glutamine metabolism has been implicated in the regulation of macrophage inflammatory phenotype in the lung. In a model of LPS-induced acute lung injury, endothelial cell-derived Rspondin3 was shown to stimulate glutaminolysis in interstitial lung macrophages, generating the TCA cycle intermediate α-ketoglutarate (α-KG), which acts as a cofactor for demethylases and thereby drives anti-inflammatory epigenetic reprogramming of interstitial lung macrophages, promoting the resolution of inflammation (109). This mechanism indicates that glutamine metabolism can modulate macrophage inflammatory phenotype switching through an α-KG–epigenetic axis. It should be noted that the glutamine–α-KG mechanism described above has been validated only in acute lung injury models; direct evidence in IPF or chronic pulmonary fibrosis remains lacking. Given that macrophages in the fibrotic lung undergo sustained metabolic reprogramming, whether glutamine metabolism participates in regulating the phenotypic balance between pro-fibrotic and anti-fibrotic macrophage populations remains an open question worthy of further investigation.

The arginine and glutamine pathways described above do not operate in isolation. They intersect at the level of the TCA cycle, and it is this convergence that gives rise to the integrated pro-fibrotic metabolic state of IPF macrophages. In the metabolic context of IPF, the imbalance of the above pathways - the enhanced pro-fibrotic metabolic flux mediated by ARG1, the weakened anti-inflammatory α-KG signal produced by glutamine breakdown, and the reduced production of itaconate derived from the TCA - collectively form the molecular basis of the pro-fibrotic metabolic reprogramming, providing a theoretical basis for the IPF treatment strategy targeting amino acid metabolism.

It is important to note that the protective effects of amino acid supplementation observed in experimental models provide a counterpoint to this pro-fibrotic metabolic picture. Functional amino acids had protective effects on apoptosis, inflammation and pulmonary fibrosis in mice stimulated by LPS. It was reported that arginine or glycine reduced the expression of IL-6 and CXCL-1 to decrease the accumulation of alveolar epithelium neutrophils and macrophages, thereby improving alveolar integrity and function, meanwhile glutamine reduced LPS-induced collagen deposition and inflammatory cytokines (110). These findings suggest that exogenous amino acid supplementation may partially restore the metabolic balance that is disrupted in the fibrotic macrophages, although direct evidence in IPF-specific macrophage populations is currently lacking.

## 4 Metabolism-related targets and pathways of macrophages regulating IPF and related pulmonary conditions

Macrophage metabolism involves multiple targets and pathways, which may be involved in regulating multiple metabolic processes at the same time. We have summarized the most critical and newly discovered targets and pathways in **Table 1**, and expounded the underlying mechanisms.

**Table 1:** Metabolism-related macrophage targets and pathways implicated in IPF and related pulmonary conditions.

Metabolism	Target	Pathway	Function in macrophage	Disease context
Glucose/TCA	Itaconate (IRG1)	Nrf2/antifibrotic axis	Endogenous antifibrotic mediator; IRG1 KO aggravates fibrosis	IPF (61)
	Itaconate (IRG1)	SDH inhibition/ETC-CI/NLRP3	Pro-inflammatory; exacerbates acute lung injury in AMs	ALI (60)
	4-OI	cGAS/STING	Inhibits alveolar macrophage pyroptosis	ALI (111)
	4-OI	PI3K/Akt/NF-κB	Anti-inflammatory; attenuates LPS-induced injury	ALI (112)
	4-OI	Nrf2	Inhibits macrophage ferroptosis via Nrf2 pathway activation	ALI (113)
	Succinate	HIF-1α/prolyl hydroxylase/NLRP3	Intracellular: stabilizes HIF-1α to drive IL-1β transcription and NLRP3 inflammasome assembly; Extracellular: engages SUCNR1 to activate PI3K/HIF-1α axis	ALI (55, 114)
	Lactate	p300/H3K18la/ARG1–PDGFA–THBS1–VEGFA	Myofibroblast-derived lactate drives histone lactylation at profibrotic gene promoters via p300 acetyltransferase, reprogramming macrophages toward pro-fibrotic phenotype	IPF (67, 69)
	Ethyl 2-Succinate-Anthraquinone	NLRP3	Attenuates inflammatory response and oxidative stress	ALI (115)
	IRF4	IL-4/STAT6; M-CSF/mTORC2; glycolytic gene regulation	Drives M2-like selective activation; regulates macrophage polarization and fibroblast activation in particle-induced fibrosis via IL-17A transcriptional activity	Cancer and fibrosis (116, 117)
	PNPT1	ROS/IKK/NF-κB/MAVS/NLRP3; HIF-1α/GLUT1/OXPHOS-glycolysis switc	Regulates mitochondrial dsRNA release; inflammasome activation; PNPT1 deficiency increases mitochondrial ROS, activates NF-κB, and induces metabolic reprogramming toward glycolysis via HIF-1α and GLUT1 upregulation	IPF-related (118, 119)
Lipid	SREBP	TLR4–NF-κB–SREBP1/IL-4–AKT–STAT6–SREBP1/DNL	Promotes *de novo* fatty acid synthesis under both pro-inflammatory (TLR4–NF-κB) and alternative activation (IL-4–AKT) stimuli; promotes lipogenesis and macrophage inflammatory activation	Chronic lung disease (91, 120, 121)
	mTORC1–SREBP1	mTORC1/lipogenic program	Activates SREBP1 to promote lipid synthesis and storage; constitutive driver of lipogenesis in nutrient-rich alveolar macrophage environment; bridges amino acid sensing (leucine/SLC7A5) and lipid anabolic programs	Chronic lung disease/IPF (122, 123)
	SCD1	PI3K-Akt-mTOR-SREBP1	Drives lipid remodeling in airway macrophages	lung fibroblasts (124)
	LXR–ACAT-1 (Soat1)	LXR/ABCA1–ABCG1–IDOL/cholesterol esterification	LXR senses oxysterols to induce cholesterol efflux transporters; when efflux is overwhelmed, ACAT-1 esterifies excess cholesterol into lipid droplets, driving foam cell transition; ACAT-1 inhibitor K-604 reduces pulmonary inflammation in bleomycin model	IPF/Lung injury (125)
	NOX4	ROS/mitochondrial biogenesis	Reduces pro-fibrotic molecule production	Asbestosis (126)
	miR-33	ABCA1/SREBP intron feedback/FAO/NLRP3 inflammasome	Suppresses cholesterol efflux via ABCA1 targeting; simultaneously impairs FAO and disrupts glycolysis–OXPHOS balance; activates NLRP3 inflammasome; creates dual metabolic block favoring lipid retention and inflammatory polarization	IPF (75, 127–132)
	PM2.5	miR-195/SREBP1/FAS	Induces lipid droplet accumulation	Environmental injury (133)
Amino Acid	Arginine	ARG1/ornithine–proline–polyamine branch-point	Provides proline substrate for fibroblast collagen synthesis; drives pro-fibrotic ECM deposition; ornithine also feeds polyamine biosynthesis branch supporting epithelial proliferation; flux distribution between branches may determine fibrotic progression or regression	IPF/Lung fibrosis (102, 104, 134)
	Arginine	iNOS/ARG1–iNOS substrate competition	IFNγ-induced NO production; pro-inflammatory and anti-bacterial; upregulated in fibrotic lung macrophages; ARG1 dominance diverts arginine flux away from iNOS, depletes extracellular arginine, inhibits T cell proliferation and limits anti-fibrotic immune response	COPD and lung fibrosis (105, 135)
	GS	HIF-1α	Supports pro-fibrotic, immunosuppressive macrophage phenotype	IPF (108)
	Glutamine	GLS/GDH–transaminase/Rspondin3/α-KG	Glutaminase (GLS) hydrolyzes glutamine to glutamate; converted to α-KG via GDH or transaminase for TCA anaplerosis; Co-factor for TET dioxygenases and histone demethylases; promotes anti-inflammatory epigenetic reprogramming of interstitial macrophage via Rspondin3-driven paracrine signaling from endothelial cells	ALI (109)
	SLC3A2/SLC1A5 (CD98)	Glutamine transporter/α-KG–epigenetic axis	Gates intracellular glutamine availability in lung macrophages; reduced transporter expression limits glutamine uptake, attenuates α-KG generation, and impairs anti-inflammatory epigenetic reprogramming capacity	IPF-relevant (92)
	SLC3A2/SLC7A5	Leucine transport/mTORC1 activation	Mediates import of large neutral amino acids including leucine; activates mTORC1 to coordinate macrophage anabolic programs including lipid biosynthesis via SREBP1, self-renewal signaling, and pro-inflammatory cytokine production; bridges amino acid and lipid metabolic axes	IPF-relevant (92, 136–138)

### 4.1 Glycolysis-related mechanisms

The downstream signaling mechanisms by which succinate, itaconate and lactate transduce metabolic changes into fibrotic and inflammatory responses involve distinct receptor interactions, enzyme modifications and transcriptional cascades. Building on the succinate accumulation described in Section 3.1, succinate acts as an extracellular and intracellular signaling molecule through distinct receptor-mediated and enzyme-inhibitory mechanisms. Intracellularly, succinate stabilizes HIF-1α by inhibiting prolyl hydroxylases, driving downstream IL-1β transcription and NLRP3 inflammasome assembly (55). Extracellularly, succinate engages SUCNR1 to activate the PI3K/HIF-1α axis, promoting macrophage polarization and cancer-associated metastatic signaling (114). At the pharmacological level, ethyl 2-succinate-anthraquinone has been reported to inhibit oxidative stress and THP-1 macrophage migration via NLRP3 pathway suppression (115). GS inhibition promotes an M1 phenotype via HIF-1α activation, which may be further stabilized by succinate accumulation in this signaling context (139).

The itaconate that accumulates during TCA reprogramming (Section 3.1) exerts its downstream effects through direct molecular interactions with key signaling nodes. The 4-octyl itaconate (4-OI) derivative rescues mitochondrial dysfunction and inhibits NLRP3-mediated macrophage pyroptosis in a STING/IRF3-dependent manner (111). 4-OI also alleviates acute lung injury by suppressing ROS-mediated PI3K/Akt/NF-κB signaling (112) and inhibits macrophage ferroptosis via Nuclear Factor Erythroid 2-Related Factor 2 (Nrf2) pathway activation (113). At the same time, as a macrophage-derived immunometabolism substance, itaconate potentially regulates the KEAP1-NRF2 pathway and the balance of glycolysis and oxidative phosphorylation in macrophages, thereby inhibiting the process of pulmonary fibrosis (61).

Within macrophages, lactate-driven histone lactylation represents the molecular mechanism linking metabolic dysregulation to epigenetic reprogramming. Kla occurs on multiple lysine residues of core histones H2A, H2B, H3, and H4; among these, histone H3 lysine 18 lactylation (H3K18la) has been identified as a functionally critical site that accumulates during macrophage polarization and directly drives transcriptional activation of target genes (69). The p300 acetyltransferase serves as the primary lactyl-transferase, catalyzing site-specific H3K18la at the promoter regions of profibrotic mediators, including ARG1, PDGFA, THBS1, and VEGFA. This epigenetic modification induces chromatin relaxation and robustly activates transcription of these profibrotic target genes, thereby reinforcing the profibrotic macrophage phenotype. Crucially, knockdown of p300 significantly attenuates both global histone lactylation and the transcriptional induction of these profibrotic mediators, confirming that this reprogramming effect is strictly dependent on p300-mediated H3K18la (67). Through the lactate/p300/H3K18la/ARG1–PDGFA–THBS1–VEGFA axis, myofibroblast-derived lactate enables site-specific epigenetic reprogramming of neighboring macrophages within the IPF fibrotic niche. This pathway directly links metabolic dysregulation to profibrotic phenotype output, representing a mechanistically defined and therapeutically actionable target for interrupting the macrophage– myofibroblast crosstalk that sustains fibrotic progression.

IRF4 is expressed in M2 macrophages and has been proved to regulate the expression of glycolytic genes (140–142). It has been found that IRF4 is involved in particle-induced pulmonary fibrosis silica by regulating macrophage polarization and fibroblast activation. IRF4 downregulation inhibits the macrophage polarization and pro-fibrotic factors release, and at the same time reduces the activation of fibroblasts by regulating the transcriptional activity of IL-17A, thus delaying the transition from early inflammatory response to fibrotic response in lung tissue of silica-exposed mice (117). The expression of IRF4 in M2 macrophages depends on IL-4/Stat6 and M-CSF/mTORC2 signaling regulation. The selective activation of macrophages by IRF4 may be regulated by mTORC2-IRF4 mediated reprogramming of glucose metabolism in macrophages. The specific mechanism remains to be explored (116).

### 4.2 ROS-mediated mechanisms

Polyribonucleotide nucleotidyltransferase 1 (PNPT1) is mainly located in the mitochondrial intermembrane space and is related to RNA degradation, processing and polyadenylation (143). Genome-wide association analysis discovered PNPT1 as candidate gene of angiotensin-converting enzyme inhibitors-induced cough and suggested that PNPT1 inhibition may mediate airway inflammation and stimulation through regulation of mitochondrial ROS production (144). PNPT1 deficiency may affect mitochondrial function, and then induce inflammatory and immune responses, which may play a potential role in lung inflammatory response and tumor immunity (145–147). PNPT1 deficiency increased ROS in mitochondria to regulate IKK phosphatase activated NF-κB signaling, so as to promote the release of inflammatory cytokines (143). Other studies have shown that PNPT 1 knockout induces metabolic reprogramming by reducing OXPHOS and increasing glycolysis (119). In contrast, PNPT1 mediates NLRP3 inflammasome activation through MAVS and metabolic reprogramming, which is independent of NF-κB (Nuclear Factor Kappa B) pathway (118). PNPT1 deficiency may also increase glycolysis by altering the expression of glucose metabolism genes HIF-1α and glucose transporter 1 (GLUT1).

Sterol regulatory element binding protein 1α(SREBP-1α) expression was increased in LPS-activated BMDM to activate fat regeneration and core inflammasome component gene NLRP1α (148). Sterol regulatory element binding protein (SREBP) is activated in response to a typical T helper cell 2 cytokine IL-4 to trigger the *de novo* lipogenesis (DNL) program that raises ROS levels and promote macrophage alternative activation (120). TGF-β1 was found to be involved in regulating SCD1 expression and lung fibroblast activation through the PI3K-Akt-mTOR-SREBP1 pathway in asthmatic airway remodeling mice (124). In a recent study on pulmonary fibrosis and LUAD that is related to lipid droplet accumulation induced by PM2.5 exposure, Zhu et demonstrated the new mechanisms based on d-limonene/miR-195/SERBP1/FAS axis that could become potential preventive intervention (133).

NADPH oxidase 4 (NOX4) is a source of cellular superoxide anions that promote the pro-fibrotic polarization of lung macrophages and increase the production of pro-fibrotic molecules that induce collagen deposition (126). Mechanistically, NOX4 further increases mitochondrial ROS production and induces mitochondrial biogenesis. Targeting redox signaling and mitochondrial biogenesis can prevent pro-fibrotic polarization in lung macrophages by reducing the production of pro-fibrotic molecules.

### 4.3 Lipid metabolism-related mechanisms

Lipid dysregulation in IPF-associated macrophages arises not from a single pathway failure but from the concurrent disruption of four interrelated processes: *de novo* lipid synthesis, cholesterol sensing and esterification, lipid storage, and cholesterol efflux.

The accumulation of lipids in alveolar macrophages is not a passive process but is caused by the imbalance of the four dynamic processes of lipid uptake, synthesis, storage and excretion. *De novo* FAS is initiated in the cytoplasm, where acetyl-CoA carboxylase 1 (ACC1) converts acetyl-CoA to malonyl-CoA, and FAS catalyzes *de novo* synthesis of palmitate in the cytoplasm, which can be further elongated in the endoplasmic reticulum (ER) by elongase*s* (149, 150).This FAS pathway is transcriptionally regulated by SREBP1, which is itself activated downstream of the TLR4–NF-κB signaling axis in response to saturated fatty acids, thereby linking pro-inflammatory stimuli to enhanced lipogenesis in macrophages (121, 148, 151). In contrast, IL-4 stimulation promotes SREBP1-dependent FAS through AKT phosphorylation and STAT6 signaling, and this lipogenic program is required for alternative macrophage activation, suggesting that FAS supports both inflammatory and anti-inflammatory polarization states depending on the upstream signal (120). Critically, mTOR complex 1 (mTORC1) activates SREBP1 and controls the expression of lipogenic binding proteins to promote lipid synthesis and storage; given that alveolar macrophages reside in a nutrient-abundant lipid-rich environment, sustained mTORC1 activity may represent a constitutive driver of lipogenesis in this cell population (122, 123). Together, the TLR4–NF-κB–SREBP1 and IL-4–AKT–SREBP1 axes, both amplified by mTORC1, indicate that *de novo* FAS is not a bystander process but an active participant in both pro-inflammatory and pro-fibrotic macrophage programs.

The LXR signaling axis operates principally as a cholesterol sensor: oxysterols generated during phagocytosis of oxidized lipids activate LXR, which in turn induces transcription of the cholesterol efflux transporters ABCA1 and ABCG1 as well as the inducible degrader of the LDL receptor (IDOL), collectively opposing intracellular cholesterol accumulation (152–154). When this efflux capacity is overwhelmed, excess free cholesterol undergoes esterification by acyl-CoA: cholesterol acyltransferase 1 (ACAT-1/Soat1), the principal cholesterol esterification enzyme in macrophages, forming cholesterol esters that are stored as lipid droplets — a process that, while cytoprotective in the short term, drives the transition to a foam cell phenotype (125, 155–159). In a bleomycin lung injury model, intratracheal administration of the ACAT-1 inhibitor K-604 reduced pulmonary inflammation, providing direct experimental evidence that ACAT-1-mediated cholesterol esterification contributes to macrophage-driven lung injury (125). This LXR–ACAT-1 axis therefore defines a critical tipping point that when LXR-driven efflux is insufficient to clear incoming cholesterol, ACAT-1 redirects free cholesterol into esterified storage, locking macrophages into a lipid-laden, pro-fibrotic state.

The transcriptional programs described above are subject to an additional layer of post-transcriptional control mediated by non-coding RNAs, among which miR-33 has emerged as a central integrator linking lipogenic transcription to cholesterol efflux suppression. MicroRNAs are involved in regulating the formation of lipid-loaded macrophages (foam cells) in the lungs. MiR-33 family is encoded within the introns of SREBP genes, positioning it as an intrinsic feedback component of the lipogenic program. MiR-33 controls cellular lipid metabolism in macrophages and inhibits expression of genes involved in cholesterol efflux (130), high-density lipoprotein biogenesis (160)and FAO (161). By simultaneously suppressing cholesterol efflux and FAO, miR-33 creates a dual metabolic block that favors intracellular lipid retention. More specifically, miR-33 targets the 3′-untranslated region of ABCA1, suppressing its expression and thereby reducing reverse cholesterol transport in macrophage foam cells, a mechanism that may contribute to lipid accumulation in the fibrotic lung (127–130). Beyond lipid transport, miR-33-mediated disruption of balance between aerobic glycolysis and mitochondrial OXPHOS could induce inflammatory polarization of macrophages and form innate and adaptive immune responses (131). MiR-33 was also confirmed to be a positive regulator of NLRP3 inflammasome in macrophages (132). Although the majority of this evidence derives from cardiovascular research, the convergent roles of miR-33 in suppressing cholesterol efflux, impairing FAO, disrupting metabolic balance, and activating the NLRP3 inflammasome suggest that it may exert equivalent regulatory influence over lipid homeostasis in alveolar macrophages in the IPF context (75). Therefore, therapeutic targeting intervention of miR-33 through means such as anti-miR oligonucleotides may become a translatable strategy for restoring lipid efflux and alleviating the progression of pulmonary fibrosis driven by foam cells.

### 4.4 Amino acid metabolism-related mechanisms

Ornithine generated by ARG1-expressing macrophages serves as a shared precursor for two downstream metabolic branches: one leading to polyamine biosynthesis, which supports local epithelial cell proliferation, and the other yielding proline, which is incorporated into collagen by activated fibroblasts, thus linking macrophage arginine catabolism directly to ECM accumulation (104, 134). The metabolic flux distribution between the two branches may be a key factor determining the progression or regression of fibrosis, but currently there is a lack of direct flux measurement data in human samples of IPF.

The third branch of arginine metabolism is mediated by the inducible nitric oxide synthase (iNOS/NOS2). Under the stimulation of IFNγ signal, arginine is oxidized to nitric oxide (NO) and citrulline. From the perspective of substrate competition, ARG1 and iNOS jointly compete for the arginine substrate. The expression ratio and relative strength of the enzymes determine the directional diversion of arginine metabolism. In fibrotic macrophages, the dominance of the ARG1 pathway can lead to a shift of arginine towards the ornithine-proline axis; at the same time, the consumption of arginine can also reduce the extracellular arginine concentration, inhibit T cell proliferation, indirectly promote immune tolerance, and limit the anti-fibrotic immune response (135). This dual consequence of ARG1 dominance — enhanced collagen substrate supply and suppressed anti-fibrotic T cell activity through arginine depletion — positions the ARG1/iNOS ratio as a mechanistically important parameter in IPF macrophage biology. Strategies that restore iNOS activity or relieve arginine depletion in the fibrotic niche may therefore have the potential to partially reverse both the fibroblast-activating and the immunosuppressive consequences of ARG1 dominance.

Glutamine is catalyzed by GS to undergo hydrolysis into glutamate. Subsequently, it is converted into α-KG through glutamate dehydrogenase (GDH) or transaminase, thereby replenishing the intermediate products of the TCA cycle (anaplerosis). In the ALI model, there is direct evidence indicating that this pathway can regulate the inflammatory phenotype of pulmonary macrophages: the angiopoietin-like protein Rspondin3 secreted by endothelial cells can activate the breakdown of glutamine in interstitial lung macrophages. The resulting α-KG acts as a cofactor for TET dioxygenases and histone demethylases, mediating DNA demethylation and histone demethylation, and driving the epigenetic reprogramming of macrophages towards an anti-inflammatory phenotype (109). This Rspondin3–GLS–α-KG–TET/demethylase cascade therefore defines a paracrine mechanism by which endothelial cells communicate metabolic and epigenetic instructions to adjacent macrophages, coupling the vascular microenvironment to macrophage phenotypic state. Whether an equivalent endothelial–macrophage glutamine signaling axis operates in the remodeled vasculature of the fibrotic lung, and whether it is impaired in IPF, represents a question of direct mechanistic relevance.

The capacity of macrophages to execute this glutamine–α-KG axis is gated at the level of membrane transport, making transporter expression a critical upstream determinant of downstream epigenetic output. At the transporter level, glutamine import in lung macrophages depends on CD98-associated carrier complexes, including the SLC3A2/SLC1A5 heterodimer; surface expression of this transporter on lung macrophages positions as a potential regulator of intracellular glutamine availability and downstream α-KG production (92). Reduced expression or activity of SLC3A2/SLC1A5 in pro-fibrotic macrophages would therefore be expected to limit glutamine uptake, attenuate α-KG generation, and consequently impair the epigenetic reprogramming capacity that supports anti-inflammatory phenotype switching — providing a transporter-level mechanism by which the fibrotic microenvironment could sustain macrophage pro-fibrotic commitment.

Beyond glutamine, a functionally distinct amino acid transport axis operates in parallel to link extracellular amino acid availability to macrophage anabolic and inflammatory programming through mTORC1. A functionally related transporter complex, SLC3A2/SLC7A5, mediates the import of large neutral amino acids including leucine, thereby supplying a key activating signal for mTORC1; activated mTORC1 subsequently coordinates macrophage anabolic programs encompassing lipid biosynthesis via sterol regulatory element-binding protein 1, self-renewal signaling, and pro-inflammatory cytokine production (92, 136–138). It is important to note that mTORC1 is a nodal integrator that connects amino acid availability to lipid anabolic programs, effectively bridging the amino acid and lipid metabolic axes within the same macrophage.

## 5 New therapeutic approach of idiopathic pulmonary fibrosis targeting systemic/macrophage metabolism

Currently, the treatment landscape for IPF is transitioning from broad-spectrum interventions to treatments with more defined molecular targets. As illustrated in **Table 2**, the new drugs that have been approved or are currently under development mainly target the widely involved inflammatory pathways or fibroblast proliferation. Some of these candidate drugs may have pleiotropic effects and participate in metabolic regulation. For instance, Nerandomilast is categorized primarily as a selective PDE4B inhibitor which reduces the infiltration of macrophages and T lymphocytes and the deposition of collagen by regulating the cAMP signaling pathway and the TGF-β1-Smad/non-SMAD (including PI3K/AKT, etc.) signaling pathway (10). Meanwhile, its elevation of intracellular cAMP also engages metabolic regulatory nodes beyond immunosuppression, and cAMP itself acts as a critical regulator in macrophage energy homeostasis. Specifically, the elevated cAMP can activate protein kinase A (PKA), which in turn inhibits AMP-activated protein kinase (AMPK) (162, 163). Within macrophages, activated AMPK signaling promotes FAO and mitochondrial function, thereby enhancing the anti-inflammatory capacity of M2 macrophages (164). Conversely, PKA-mediated inhibition of AMPK downstream of cAMP may attenuate these metabolic adaptations, suggesting that Nerandomilast’s immunomodulatory effects could extend to macrophage bioenergetic reprogramming. This could potentially serve as a promising direction for further research to explore Nerandomilast’s pleiotropic effects in cellular metabolic homeostasis.

**Table 2:** Summary of clinical and experimental therapeutic agents for IPF.

Drug/agent	Target	Primary category	Macrophage specificity	Phase/status (for IPF)
Nintedanib	Tyrosine kinases (VEGFR, FGFR, PDGFR)	Anti-fibrotic	Not macrophage-specific; primarily targets fibroblasts and endothelial cells	FDA Approved
Pirfenidone	TGF-β1, TNF-α, Collagen synthesis	Anti-fibrotic*	Pleiotropic; indirect modulation of macrophage activation reported but not mechanistically isolated	FDA Approved
Nerandomilast	PDE4B	Anti-inflammatory*	PDE4B expressed in macrophages; alveolar macrophage subset selectivity unconfirmed in IPF	FDA Approved
Pamrevlumab	CTGF	Anti-fibrotic	Not macrophage-specific; primarily targets fibroblast-ECM axis	Phase III (165)
BMS-986278	LPA1 receptor	Anti-fibrotic/Anti-inflammatory	LPA1 expressed on macrophages, but macrophage metabolic effects not defined in trial context	Phase II (166)
Metformin	AMPK/Mitochondrial Complex I	Broad Metabolic†	Systemic AMPK activator; multi-tissue effects; no IPF-specific evidence for alveolar macrophage-selective metabolic targeting	Clinical/Pre-clinical (167)
Dimethyl Fumarate	Nrf2/Redox balance	Broad Metabolic†	Nrf2 activation occurs across multiple cell types; macrophage-specific antifibrotic effect suggested but not cell-isolated	Pre-clinical (168)
2-Deoxy-D-glucose (2-DG)	Hexokinase/Glycolysis	Broad Metabolic†	Inhibits glycolysis globally; pre-clinical evidence primarily in IPF fibroblasts; macrophage therapeutic window undefined	Pre-clinical (169)
MitoQ/SkQ1	Mitochondrial ROS	Broad Metabolic†	Broad cellular distribution; macrophage-specific ROS modulation in IPF not directly validated	Pre-clinical (170, 171)
Itaconate analogues (e.g., 4-OI)	IRG1/Nrf2/SDH inhibition	Macrophage-enriched Metabolic‡	IRG1 expression enriched in macrophages; 4-OI demonstrated macrophage-specific antifibrotic reprogramming in IPF models; strongest macrophage-centric mechanistic evidence among metabolic agents	Pre-clinical (61)

Primary Category reflects the dominant pharmacological mechanism identified in clinical or pre-clinical studies. *Pleiotropic agents where anti-inflammatory and anti-fibrotic actions overlap and cannot be fully attributed to a single mechanism or cell type. †Broad Metabolic: exerts systemic or multi-tissue effects; evidence in IPF is predominantly from bleomycin models or non-IPF contexts; no validated macrophage-selective targeting in human IPF for any agent in this category. ‡Macrophage-enriched Metabolic: preferential or functionally validated activity in macrophages within lung fibrosis context; itaconate analogues are supported by human IPF BAL macrophage data, though no clinical trials have been initiated.

It should be noted that the systemic administration of these anti-inflammatory and anti-fibrotic agents often leads to off-target effects and impaired host defense (10). In contrast, metabolic reprogramming aims to ‘re-educate’ rather than ‘suppress’ macrophages. By selectively modulating the metabolic checkpoints identified in previous sections, such as the PFKFB3-driven glycolytic pathway or the succinate-HIF-1α axis, it offers a promising avenue to precisely re-polarize Mo-AMs from a pro-fibrotic to a pro-resolving phenotype, effectively bypassing the systemic side effects associated with broad immunosuppression (90). However, there is still limited direct evidence in the pulmonary macrophages of patients with IPF, and further experiments are needed for verification.

Furthermore, a critical distinction must be drawn regarding the clinical readiness of these therapies. While anti-fibrotic and anti-inflammatory agents are largely in advanced clinical phases (Phase III), except for re-purposed drugs like metformin, the emerging metabolic regulators in the field of IPF are still mainly in the preclinical stage. The challenge for these metabolic candidates lies not only in proving efficacy within the complex fibrotic microenvironment but also in achieving lung-specific delivery to avoid systemic metabolic interference.

To this end, based on the effects of metabolic reprogramming on the plasticity and function of macrophages, therapies targeting the metabolic pathways of AMs may help develop new strategies for IPF. In the field of tumor therapy, targeting metabolic characteristics has become a new direction of tumor immunotherapy, which can restore macrophage immune function by interfering with TAMs metabolism. The treatment of IPF can also refer to the same treatment strategy.

Overactivated pro-inflammatory macrophages often contribute to the progression of inflammatory diseases, such as atherosclerosis and obesity. However, the polarization of macrophages is not fixed but rather undergoes transitions along a continuous phenotypic spectrum in response to constantly changing microenvironmental signals; there are significant differences in the dominant macrophage subpopulations at different stages of disease onset and progression, and their metabolic programs also change - from a pro-inflammatory state dependent on glycolysis to an anti-inflammatory or pro-fibrotic phenotype dependent on OXPHOS. This stage-dependent plasticity makes therapeutic strategies that target macrophage metabolism to interfere with IPF a formidable challenge.

On the one hand, new therapies can focus on reducing the number or function of pro-inflammatory or pro-fibrotic macrophages, or enhancing the anti-inflammatory, anti-fibrotic, and resolution-promoting properties of macrophages. Glycolytic inhibition could be the most important and effective anti-fibrosis strategy in the treatment of IPF. Serum amyloid P has been shown to inhibit the accumulation of pro-fibrotic macrophages in lungs of bleomycin-induced mice, suggesting its developmental prospect as a therapeutic strategy for IPF (172). Besides, alendronate inhalation has also been shown to ameliorate emphysema in mice by inducing apoptosis of AMs (173). Adoptive-transfer and transplantation techniques employing pulmonary macrophages are novel approaches being investigated as strategies for increasing the number of restorative macrophages (174). Pulmonary macrophage transplantation (PMT) is a promising new cell therapy that have been evaluated for hereditary pulmonary alveolar proteinosis (hPAP), suggesting its potential development in IPF treatment (175). Several distinct therapeutic approaches that lead to changes in the number of macrophages are being investigated.

On the other hand, the therapeutic potential of macrophages can be enhanced by developing specific macrophage targeting delivery systems. Integrating nanodelivery systems with longitudinal real-time *in vivo* tracing technology holds promise for detecting macrophage metabolic targets across successive stages of IPF progression and achieving targeted delivery of intervention drugs matched to the predominant macrophage phenotype at each stage. Recent evidence supports this approach: CeO2@PLP nanoparticles, engineered with TREM2-mediated lung targeting, have demonstrated that simultaneous lactate scavenging and macrophage metabolic reprogramming can attenuate fibrosis by suppressing pathological M2 polarization (64). For another example, the miR-33 ablation would be a novel promitochondrial anti-miRNA therapy for IPF, however, therapeutic strategies for anti-miRNA technologies are often hampered by physiological and cellular barriers to delivery to target cells (176). A technological breakthrough in nanoparticles deliveries would promote the development of specific antisense inhibitor of miR-33 targeting lung macrophage as novel therapeutic approach for IPF.

It should be noted that many of the mechanistic evidence reviewed in this article originated from the mouse lung fibrosis model induced by bleomycin. The bleomycin-induced mouse model produces an acute, self-limiting lung injury with partial spontaneous resolution, which contrasts with the chronic, progressive, and irreversible fibrotic remodeling that defines human IPF. Although this model has played a significant role in elucidating the metabolic reprogramming of macrophages, it would not fully recapitulate the progressive nature of human IPF. Furthermore, bleomycin itself has direct cytotoxicity and may independently affect the recruitment pattern of macrophages. It cannot be used to explain the genetic-related pathogenesis of IPF (such as telomerase mutations, MUC5B variations, etc.). Besides, there are species differences in the polarization lineages of macrophages. The well-defined M2-type markers (such as Arg1) that are characteristic in mice are expressed to a limited extent in human macrophages, which makes the direct phenotypic transformation more complicated.

Taken together, the burgeoning field of macrophage metabolic reprogramming represents a paradigm shift in the treatment of IPF, moving beyond broad-spectrum immune suppression toward the precise ‘re-education’ of cellular function. By decoding the bioenergetic mechanisms that govern macrophage plasticity, researchers can explore strategies to transition these cells from pro-fibrotic drivers to facilitators of tissue repair. While the spatiotemporal heterogeneity of metabolic phenotypes remains a significant challenge, the integration of targeted metabolic modulators with advanced delivery systems offers a viable therapeutic horizon. As the gap between metabolic insights and clinical application narrows, these strategies hold the potential to attenuate the progressive fibrotic remodeling and fundamentally improve the preservation of lung function and long-term clinical outcomes.
